# Reductively PEGylated carbon nanomaterials and their use to nucleate 3D protein crystals: a comparison of dimensionality†
†Electronic supplementary information (ESI) available: Experimental details, characterization and example calculations. See DOI: 10.1039/c5sc03595c


**DOI:** 10.1039/c5sc03595c

**Published:** 2016-01-29

**Authors:** Hannah S. Leese, Lata Govada, Emmanuel Saridakis, Sahir Khurshid, Robert Menzel, Takuya Morishita, Adam J. Clancy, Edward. R. White, Naomi E. Chayen, Milo S. P. Shaffer

**Affiliations:** a Department of Chemistry , Imperial College London , London SW7 2AZ , UK . Email: m.shaffer@imperial.ac.uk; b Computational and Systems Medicine , Department of Surgery and Cancer , Imperial College London , London SW7 2AZ , UK . Email: n.chayen@imperial.ac.uk; c Laboratory of Structural and Supramolecular Chemistry , Institute of Nanoscience and Nanotechnology , National Centre for Scientific Research ‘Demokritos’ , Athens , Greece; d Toyota Central R&D Labs., Inc. , Nagakute , Aichi 480-1192 , Japan

## Abstract

A range of carbon nanomaterials, with varying dimensionality, were dispersed by a non-damaging and versatile chemical reduction route, and subsequently grafted by reaction with methoxy polyethylene glycol (mPEG) monobromides. The use of carbon nanomaterials with different geometries provides both a systematic comparison of surface modification chemistry and the opportunity to study factors affecting specific applications. Multi-walled carbon nanotubes, single-walled carbon nanotubes, graphite nanoplatelets, exfoliated few layer graphite and carbon black were functionalized with mPEG-Br, yielding grafting ratios relative to the nanocarbon framework between *ca.* 7 and 135 wt%; the products were characterised by Raman spectroscopy, TGA-MS, and electron microscopy. The functionalized materials were tested as nucleants by subjecting them to rigorous protein crystallization studies. Sparsely functionalized flat sheet geometries proved exceptionally effective at inducing crystallization of six proteins. This new class of nucleant, based on PEG grafted graphene-related materials, can be widely applied to promote the growth of 3D crystals suitable for X-ray crystallography. The association of the protein ferritin with functionalized exfoliated few layer graphite was directly visualized by transmission electron microscopy, illustrating the formation of ordered clusters of protein molecules critical to successful nucleation.

## Abstract 2

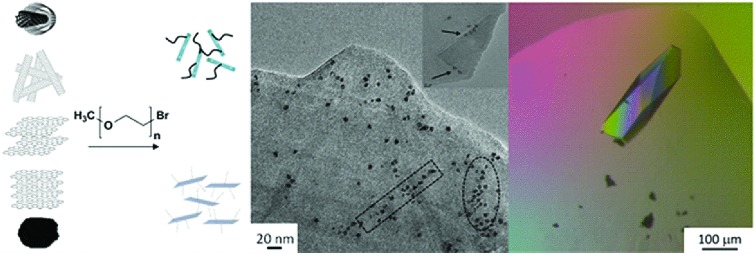
Reductive grafting with mPEG is effective on a wide range of carbon nanomaterials. However, 2D forms are most effective as protein nucleants.

## Introduction

There is a need to chemically modify carbon nanomaterials (CNMs) to improve both processing and function.^1^,^2^ A wide range of general chemistries have been explored, especially for carbon nanotubes (CNTs),^3^,^4^ including for example electrochemical,^5^,^6^ thermochemical,^7^ diazonium coupling,^8^,^9^ and reduction^10^–^12^ methods. Many of the approaches, in principle, may be applied to a range of CNMs, although very few comparative studies exist. In recent years, many established methods have been reapplied to functionalize graphene;^13^–^16^ whilst the chemistry is similar, bulk functionalization of graphene has been less straightforward, since stronger van der Waals forces and natural defects of the starting materials tend to limit exfoliation. Aggressive acid oxidation or intense sonication is often used for both graphene and CNT materials to improve individualization, at the expense of damaging the intrinsic C–C bonded framework^17^,^18^ and hence the properties of interest. An encouraging alternative is the spontaneous formation of thermodynamic solutions of undamaged carbon nanomaterials *via* charging protocols, including both reduction^10^,^11^,^15^,^19^–^22^ and oxidation^23^/protonation.^24^ Polyelectrolyte anionic forms of CNTs and graphene, now more familiarly known as nanotubide^6^,^10^ and graphenide,^15^ can be generated by reduction with alkali metals in liquid NH_3_,^5^,^14^,^25^ with alkali metals plus a charge transfer agent in organic solvent (*e.g.* naphthalide/tetrahydrofuran),^10^,^16^,^21^,^26^ or electrochemically.^6^,^27^ All reductive charging techniques insert electrons into the CNM π* orbitals or conduction band, raising the Fermi energy, and in turn increasing reactivity. This charge is then exploited for subsequent single electron transfer (SET) reactions.^20^,^26^,^28^ The degree of functionalization depends on several factors, including absolute ionic concentration,^20^ the reduction or SET potential of the reactant,^29^ charge : carbon ratio,^23^,^30^ ionic strength/dielectric constant of the medium, degree of exfoliation, and the steric bulk of the grafting agent.^19^,^20^ The versatility and non-damaging character of the chemistry allows systematic studies of both intrinsic chemistry and functionalized products for particular applications.

The availability of three dimensional protein crystals is a fundamental bottleneck limiting structure determination of target proteins relevant to future drug design. Well-designed nucleants (protein crystal nucleation-inducing substrates) aid the crystallization of new target proteins and potentially provide a deeper understanding of different protein crystallization mechanisms.^31^,^32^ It may not be the case that *one nucleant fits all*, but the versatility of carbon nanomaterial chemistry provides an exciting platform to crystallize a variety of proteins. Two previous works have considered using carbon nanomaterials as additives or nucleants for three dimensional protein crystallization: one used gelatine-coated CNT buckypapers^33^ and the other colloidal graphenes;^34^ however, there has not been a controlled and systematic study which correlates CNM chemistry and geometry with nucleation. TEM imaging studies have observed protein adsorption, ordering and/or 2D crystallization on unfunctionalized MWNTs^35^,^36^ but have not grown 3D crystals for X-ray crystallography. General nucleation studies, which have provided theoretical models for heterogeneous nucleation mechanisms, conclude that geometry and pore size are two important driving forces.^32^,^37^,^38^ A broad pore size distribution is favourable for nucleating different proteins, due to significant variations in protein size and their critical nuclei. Theoretical models have shown that pores may confine and stabilise protein molecules and in-turn encourage crystalline nuclei to form and grow.^34^,^37^ Functionalized CNMs are, therefore, interesting substrates for protein nucleation due to their high accessible surface areas and ability to form networks, heterogeneous in both topography and chemistry, providing a broad distribution of ‘pockets’ in which proteins may accumulate. Chemical modification enhances compatibility and solubility with (usually aqueous) protein conditions. Although this study focuses on one family of chemical functionality (polyethylene glycol), it is possible to graft a variety of functionalities, providing a versatile methodology to adjust geometry and chemistry for crystallizing proteins.

The aim is to design nucleants which encourage protein nucleation deep into metastable supersaturated solutions (promoting single crystal growth) and at low protein concentrations. This study uses reduction chemistry to graft methoxy poly(ethylene glycol) (mPEG) on an array of carbon nanomaterials. mPEG was selected following preliminary trials and its particular effectiveness in grafted form is intriguing since free PEGs are already used in many protein crystallization conditions. PEG-modified CNMs, especially CNTs, are also of interest for biological applications, such as drug delivery^39^,^40^ and biosensing,^41^,^42^ but have generally been prepared *via* either non-covalent methods^43^ or coupling to acid-oxidized materials.^44^,^45^ This study provides an exploration of reductive chemistry to graft mPEGs to a variety of CNMs, specifically including multi-walled carbon nanotubes (MWNTs), single-walled carbon nanotubes (SWNTs), exfoliated few layer graphite (FLG), graphite nanoplatelets (GNPs) and carbon black (CB), providing a panel of materials to assess systematically the effect of geometry on both reductive functionalization and protein crystallization. These materials provide a full range of dimensionality FLG (2D), SWNT (1D, high curvature), MWNT (1D, low curvature), and CB (0D), with broadly similar chemistry.

## Results and discussion

### PEGylated carbon nanomaterials *via* reductive chemistry

Starting materials were sourced from commercial suppliers: graphite (Graphexel natural crystalline flake graphite, grade: 2369, Graphexel Ltd., UK) for exfoliation to few layer graphite, graphite nanoplatelets, likely produced by exfoliation of graphite *via* acid intercalation^46^ (XG Sciences), fluidized bed CVD-grown MWNTs (Arkema),^47^ purified SWNTs (Thomas Swan Elicarb P925) synthesized by CVD on substrate^48^ and carbon black (furnace black, Printex L6, Degussa).^49^ The as-received materials were dried and reductively charged as described in detail in the ESI.† Brominated^50^,^51^ 5 kDa mPEG (mPEG-Br) was prepared *via* a literature method^52^ (further details in the ESI†). Carbon nanotubide,^5^ graphenide^15^ and reduced carbon black solutions were functionalized with mPEG-Br, adapting routes developed for simple alkylations. In short, reduced nanocarbon solutions were prepared by mixing a pre-made sodium naphthalide solution (either in tetrahydrofuran, THF or dimethylacetamide, DMAc) with dried nanocarbon with a charging ratio (C/Na) 12/1 (for flat sheet geometries) and 20/1 (for tubular and spherical geometries). The ratio C/Na denotes the number of framework carbon atoms per sodium atom used for charging. The different ratios were selected, based on optimums identified previously for the reductive grafting of alkyl chains, which maximize exfoliation by balancing the total charge available against the tendency for charge condensation.^19^,^26^ The functionalization reaction mechanism remains a topic of debate but it is likely that the PEG free radical generated by single electron transfer from sodium naphthalenide to the PEG-Br, yields functionalized products as a result of radical–radical anion combination.^53^–^55^ PEG-functionalized samples were thoroughly washed to remove physisorbed polymer and dried under vacuum.

**Fig. 1:**
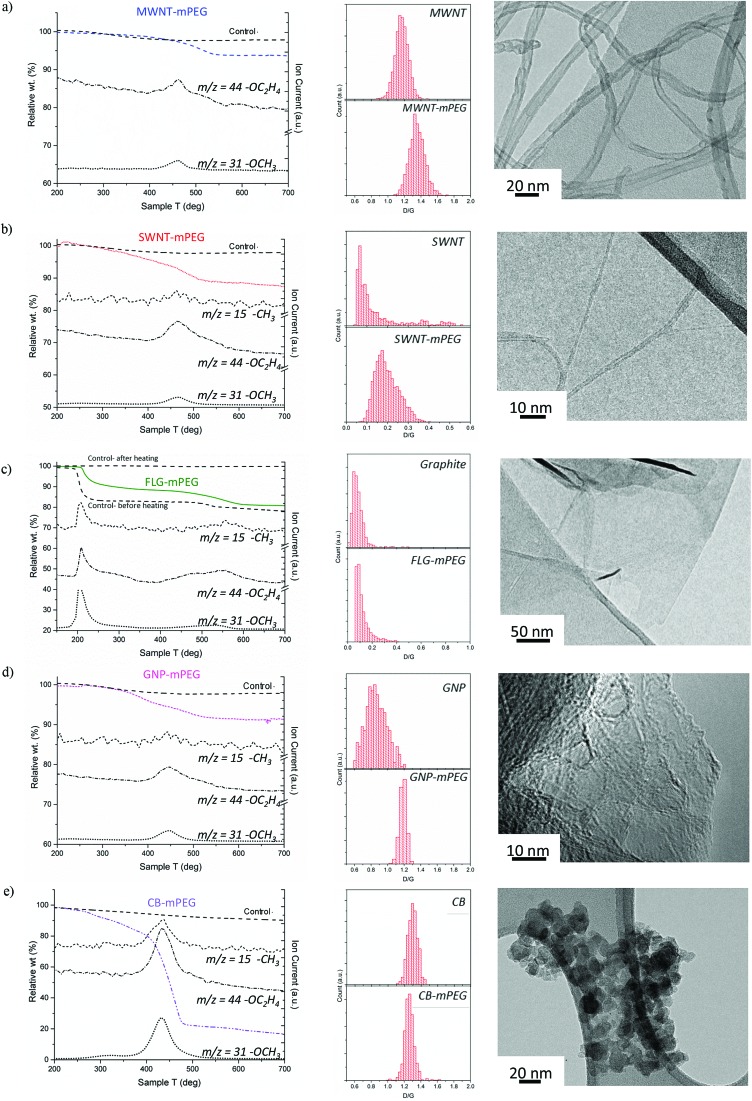
TGA-MS characterization of washed samples and controls (addition of unreactive poly(ethylene glycol) dimethyl ether), Raman mapping histograms (counts of region with given D/G) and TEM images of mPEG grafted (a) MWNTs, (b) SWNTs, (c) FLG (including before and after heating control in TGA), (d) GNPs and (e) carbon black. *m*/*z* = 15 –CH_3_^+^, *m*/*z* = 44 –CH_2_CH_2_O^+^ and *m*/*z* = 31 –OCH_3_^+^ are fragments of mPEG (see TGA-MS of pure mPEG and Raman spectra in ESI†).

Thermogravimetric analysis (TGA) under nitrogen (Fig. 1) indicates successful grafting of mPEG and was used to determine the polymer grafting ratio (wt% of grafted organics relative to initial carbon framework, Table 1) from the mass loss attributable to the decomposition of grafted mPEG (see Fig. S1a†). The functionalized MWNT, SWNT and GNP samples all display one significant weight loss between 450–550 °C; control experiments, examining samples treated equivalently using unreactive poly(ethylene glycol) dimethyl ether, showed no significant mass loss after washing, and were used as a baseline for calculating grafting ratio. Mass spectrometry showed that the mass loss in grafted samples correlates with units observed in PEG decomposition, including the monomer (*m*/*z* = 44 –C_2_H_2_O^+^), methyl (*m*/*z* = 15 –CH_3_^+^) and methoxy groups (*m*/*z* = 31 –OCH_3_^+^), although for the MWNTs, the weakest *m*/*z* = 15 feature was not observed due to the low grafting ratio. The peak degradation temperature was significantly higher (*ca.* 70 °C) for the grafted materials than the pure mPEG 5 kDa-Br reference (ESI Fig. S1b† for TGA-MS of pure mPEG control), for which the decomposition was completed by 450 °C; such shifts are often attributed to covalent grafting,^56^ or at least an intimate interaction at the interface. Similar grafted PEG decomposition features were observed for both FLG and carbon black samples; however, additional features also appeared. For FLG-mPEG, there is a clear initial step at 220–250 °C, attributed to a combination of residual polymer and THF trapped within graphene layers, as confirmed by TGA-MS data (Fig. S2†). In this case, the control sample, (Fig. 1b) shows a similar first step but a very much reduced second step; the grafting ratio was therefore calculated from the relative increase in the second step (450–600 °C). The weight loss temperature for FLG-mPEG was slightly higher than for the other nanocarbons, most likely due to the constraint of the grafted polymer trapped between layers. In the carbon black sample, there is a broader decomposition feature with an early onset around 250 °C (in this case, TGA-MS shows no significant residual solvent). Although the control sample shows no physisorption of the unreactive polymer, the relatively large and broad weight loss in CB-mPEG may include some physisorbed polymer trapped by the grafted polymer chains. High structure carbon black has a large number of primary nanoparticles fused within each aggregate (TEM in Fig. 1e), generating a highly convoluted internal pore volume.

**Table 1:** Summary of grafting ratios and further analysis of mPEG functionalized carbon nanomaterials

Nucleant material	*r*_CNM_ ^*a*^ (nm)	Grafting ratio (wt% of PEG)	Grafted stoichiometry (C : PEG)	Dispersibility (μg ml^–1^)	Dry surface area^*b*^ (m^2^ g^–1^)	Surface concentration of grafted PEG (μmol m^–2^)	PEG separation, *D* (nm)
MWNT-mPEG	5.0 ± 1.2	6.8 ± 1.5	6094	68 ± 5	180	0.076	5.3
SWNT-mPEG	0.8 ± 0.4	12.6 ± 1.1	3303	51 ± 5	670	0.038	7.5
FLG-mPEG	n/a	10.6 ± 3.2	3924	35 ± 5	10^*c*^	0.031^*d*^	8.2
GNP-mPEG	n/a	11.0 ± 2.2	3792	200 ± 5	680	0.032	8.1
CB-mPEG	6.5 ± 1.1	135.3 ± 6.1	308	100 ± 5	220	1.230	1.3

^*a*^Average CNM primary radius/local radius of curvature.

^*b*^Specific surface area measured by BET.

^*c*^Small value due to restacking sheets on drying.

^*d*^Estimated using surface area of GNP to approximate a realistic surface area in solution.

Polymer grafting ratio (wt%) can be converted into an estimate of the number of structural nanoparticle carbons per mPEG chain (C : PEG ratio, ‘grafted stoichiometry’, Table 1). MWNT-mPEG has the lowest grafting ratio and therefore highest grafted stoichiometry compared to other nanocarbons, in part, as it has the lowest specific surface area of the materials studied (∼180 m^2^ g^–1^); conversely, SWNT-mPEG has a higher proportion of grafted polymer than MWNTs most likely due to the larger accessible surface area of SWNTs (670 m^2^ g^–1^). GNPs have a slightly higher specific surface area and exhibit a similar degree of functionalization when compared to SWNTs, whereas CB has a similar specific surface area to functionalized MWNTs but shows a much higher apparent degree of functionalization. The surface concentration of grafted polymer was estimated by relating PEG concentration to CNM specific surface area (Table 1). Generally, the materials have a similar density of functionalization on their exposed surfaces, except for CB-mPEG, which is significantly greater, due either to its convoluted geometry or higher defect density.

Although the details are complex, Raman spectroscopy is widely used to provide a semi-quantitative indication of crystallinity and subsequent degree of functionalization by comparing the relative intensity (D/G) of the defect band (D-band) at ∼1350 cm^–1^ and the graphite band (G-band) at ∼1580 cm^–1^. Raman mapping provides a reliable means to assess the degree of functionalization, using the D/G ratio,^57^,^58^ including in heterogeneous materials, since it is possible to analyze thousands of independent spectra to obtain an overall statistical change before and after grafting. The Raman data generally confirms the covalent functionalization of the carbon nanomaterials through an increase in D/G ratio (Fig. 1a–e) which is known to correlate with disruption of the conjugated framework.^59^,^60^ For CB, the D/G ratio is either unchanged or slightly decreased on functionalization, as expected for extremely defective graphitic materials,^57^,^61^ for which the sensitivity is lost or even inverted.^62^ The much lower perfection for CB compared to the MWNTs is indicated by much broader and weaker Raman peaks (average Raman spectra in ESI Fig. S3†). On the other hand, average Raman spectra of FLG-mPEG generally showed a broad, shifted 2D band, with a shape consistent with the formation of functionalized bi/tri-layer graphene.^63^,^64^ In addition, single point Raman spectra of graphene layers were detected (Fig. S3†), containing a symmetrical Lorentzian 2D band of high intensity and relatively narrow band width (FWHM 55 cm^–1^); functionalized FLG layers were also observed by AFM with average flake size 1–3 μm and 1–5 nm height (see ESI, Fig. S4†). TEM observations (Fig. 1a–e) highlight the variation in geometry of the different grafted materials, and the different modes of agglomeration in the dried forms, as discussed in more detail below.

The dispersibility of each (functionalized) nanocarbon was measured by UV-Vis absorbance after sonicating in water for 10–15 minutes and centrifugation at 1000–5000*g* (see Fig. S5† for an example of FLG-mPEG in water). All materials showed improved dispersibility post-functionalization (Table 1), as expected with grafted mPEG chains. In some cases, particularly the as-received GNPs, the solubility in water was already significant (value 200 μg ml^–1^ with initial loading 1 mg ml^–1^), most likely due to oxidation during the commercial exfoliation process. Despite the mPEG functionalization, which improves water compatibility, the relatively low grafting ratio and amphiphilic character of the products lead to slow sedimentation over a period of 24–72 h at a rate of ∼6.5 μg ml^–1^ per day, (∼6% per day) as monitored by UV-Vis.

### Protein crystallization with PEGylated carbon nanomaterial nucleants

Six proteins were tested: lysozyme, thaumatin, trypsin, hemoglobin, catalase and RoAb13, to determine the nucleation properties of carbon allotropes functionalized with mPEG. The hanging drop method was used for all crystallization trials (further details can be found in the ESI† and references cited^65^,^66^). All nucleant trials were carried out at metastable conditions, namely crystallization conditions that are typically able to sustain growth but not sufficient for crystals to nucleate. The metastable conditions were determined by varying protein and crystallizing agents concentration. Protein crystallization controls were implemented to ascertain the role of unbound mPEG chains and raw carbon nanomaterials as compared to the covalently grafted mPEG-CNMs. The amount of grafted mPEG associated with the nucleants was relatively low compared to the free homopolymer PEG included in the crystallization agents. Based on the grafting ratio of mPEG on the CNMs (Table 1), and the volume of the drops, the content of grafted mPEG was estimated to be equivalent to 0.1–1% w/v PEG in the crystallization agents. Therefore, controls containing an additional 1% w/v mPEG 5 kDa homopolymer were conducted. No crystals were observed in any of these controls for any of the three proteins for >28 days. The aim when introducing the nucleants is to have as little nucleant as possible present in the crystallization drop; it is therefore advantageous to use the dispersed nucleant in liquid form, as nucleant concentration can be adjusted and diluted (the methodology is described in the ESI†).

**Fig. 2:**
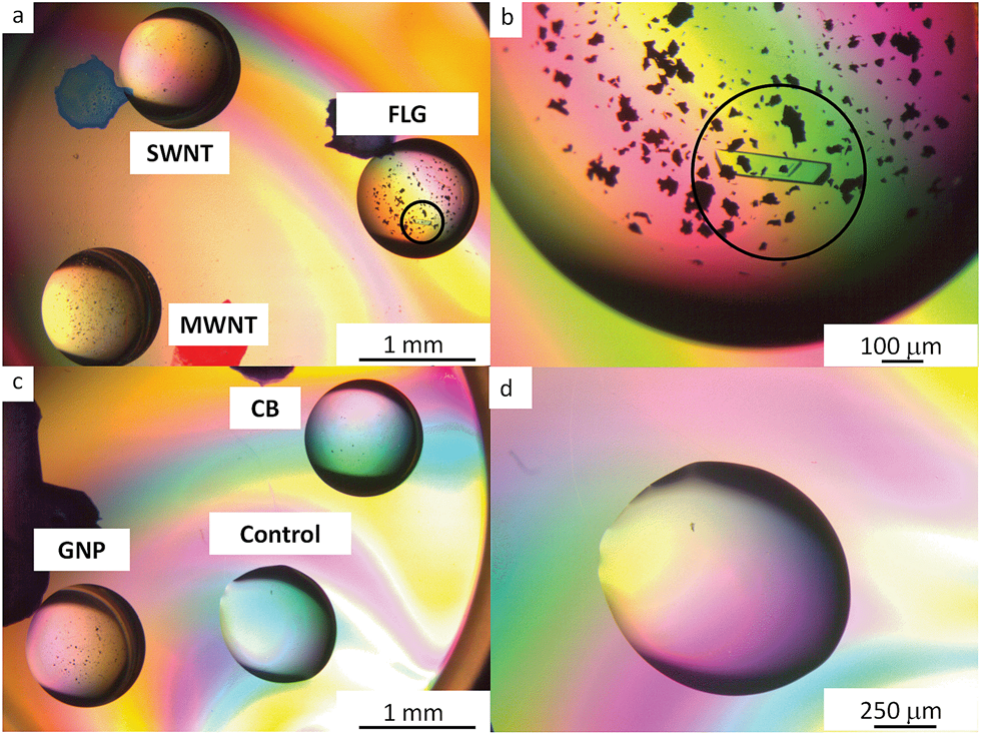
Polarized optical microscopy of 30 mg ml^–1^ trypsin drops: (a and c) containing mPEG functionalized nanocarbons: FLG, SWNTs, MWNTs, GNPs and CB (b) the circled single crystal formed within 24 h is surrounded by the FLG-mPEG flakes (d) highlights the clear control after 72 h at this protein concentration and crystallization condition. The smaller, black features are agglomerates of functionalized CNMs. Other examples of protein crystals are available in Fig. S7.†

Two liquid nucleants, FLG-mPEG and GNP-mPEG induced crystallization of thaumatin, lysozyme and trypsin at lower protein concentrations, within 48 h, compared to either no crystals or appearance only after 72 h with other nucleants (as summarized in Table 2). In comparison, all control drops (no nucleant present) were clear at these conditions. Trypsin (which is more difficult to crystallize at lower protein concentrations than lysozyme and thaumatin) did not crystallize in droplets containing functionalized MWNT, SWNT or CB nucleants. Crystallization trials with the raw carbon nanomaterials were also conducted and did not yield good crystallization results. As-received MWNTs and SWNTs crystallized lysozyme, whereas graphite crystallized thaumatin. However, the proteins only crystallized at higher protein and crystallization condition concentrations when compared to the functionalized materials. The proteins did not crystallize with as-received CB and GNP. To ensure reproducibility, FLG-mPEG and GNP-mPEG were synthesized independently five times (see Fig. S6†) and each time the product successfully and consistently crystallized proteins. In addition, the crystallization experiments were conducted over several weeks and repeated several times. In order to also test whether the amount of mPEG grafted surface was more significant than geometry, another crystallization trial was conducted, for which the PEGylated nucleant loading was adjusted to maintain a constant concentration of grafted PEG; FLG-mPEG continued to be the most effective nucleant (Fig. 2, additional crystal images are provided in Fig. S7†). In summary, compared to alternatives with similar surface chemistry, grafting density, and surface area, the flat graphene-related geometries, FLG-mPEG and GNP-mPEG, proved to be the most effective as protein nucleants.

**Table 2:** Proteins and crystal appearance times using different PEGylated nanocarbons, 1 μl dispensed into the medium as liquid nucleant dispersions

Nucleant	Crystal appearance time		
	Lysozyme	Thaumatin	Trypsin
MWNT-mPEG	*x*	*x*	*x*
SWNT-mPEG	72 h	72 h	*x*
GNP-mPEG	48 h	48 h	48 h
FLG-mPEG	48 h	48 h	48 h
CB-mPEG	*x*	*x*	*x*

To investigate the potency of the flat graphene-related nucleants further, three more proteins: hemoglobin, catalase and RoAb13 were tested (full conditions in Table S2†). Hemoglobin and RoAb13 produced single crystals with FLG-mPEG. Interestingly, catalase only crystallized with FLG-mPEG, and no other nucleants tested in this study including GNP-mPEG. In the case of catalase, to the authors' knowledge, FLG-mPEG is the only nucleant to date that has induced crystal formation at metastable conditions. Most proteins, such as thaumatin, lysozyme and trypsin form crystals from solution whilst catalase first forms a precipitate from which crystals are grown. The success of FLG-mPEG with catalase as well as with the other proteins indicates that this material may have the potential to become a more universal nucleant for protein crystallization.

### Nucleation mechanism of ferritin on FLG-mPEG

**Fig. 3:**
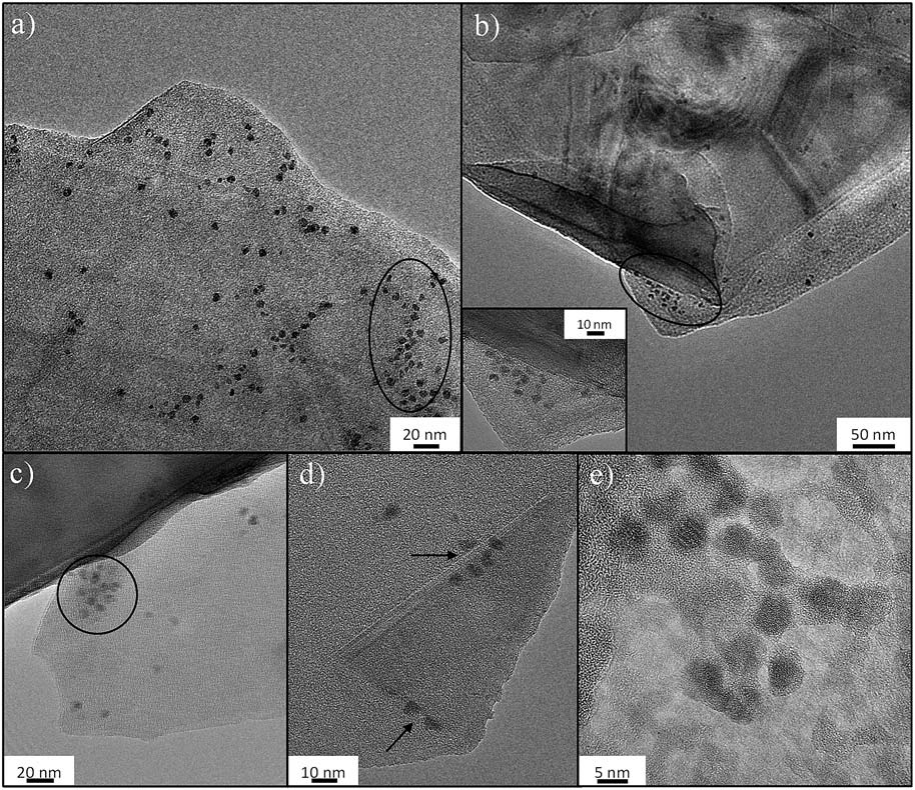
TEM images of ferritin on FLG-mPEG: (a) clusters of ferritin nanoparticles (circled) on stacked functionalized graphene layers; (b–d) ferritin at edges and creases (inset in (b) and folds as indicated by arrows in (d)); (e) higher resolution image of ferritin particles showing the lattice structure of Fe and clustering of ferritin.

The molecular basis of the protein-precipitating action of PEG is not fully understood but theoretical and experimental studies have reinforced the notion that the precipitation process caused by PEG is due primarily to an excluded volume effect.^67^ The effectiveness of PEG-grafted surfaces for nucleation is surprising, since dense PEG layers are often used to suppress protein binding;^68^ however, the low grafting density, as indicated in this work, may be critical to encourage nucleation. To further understand the nucleation process, FLG-mPEG, was combined with ferritin, as its high iron content intrinsically gives good contrast in TEM, unlike previous studies using stains.^35^,^36^ Here, the layers of functionalized FLG show concentrated areas of ferritin accumulation on the flat face as well as at sheet edges (circled in Fig. 3a–c). A corner of the graphene material is clearly seen folded in Fig. 3d with ferritin protein ordering along the edges. A control sample of ferritin combined with exfoliated but unfunctionalized FLG (see Fig. S8†) shows only a low concentration of protein agglomeration at edges but not on the surface. Thus it seems that the heterogeneous distribution of mPEG on the surface may be dictating protein segregation and assembly. The spacing, *D* between grafted 5 kDa mPEG chains on FLG is estimated to be 8.2 nm (Table 1 and ESI†); very similar values have been reported^69^–^71^ for the Flory radius, *R*_F_ (6.3–9.1 nm), of PEG 5 kDa. de Gennes' model proposes that when *D* > *R*_F_ the polymer conformation follows a mushroom regime.^72^ It appears that, in this ‘graft-to’ reaction, PEG addition is limited once the chains begin to interact, leaving a rather sparsely modified surface, unlike the dense PEG brushes used to suppress protein adsorption.^73^,^74^ The average spacing between the chains is larger than the diameters of the proteins nucleated (the average protein hydrodynamic diameters of lysozyme,^75^ trypsin^76^ and thaumatin are 1.8 nm, 1.9 nm and 3 nm, respectively), suggesting that there should be regions where proteins can be accommodated. It is therefore likely that the spacing and PEG conformation on the flat surface structure (*i.e.* average pore size and specific surface area Tables S1† and 1) contributes to the stabilization of protein nuclei. All the materials, except the graphene-related nucleants, have a radius of curvature similar to the proteins. Thus any ordered array of adsorbed protein molecules also has significant curvature. Such a curved packing is not consistent with infinite 3D translational symmetry and thus does not nucleate 3D crystals readily, instead forming a helical structure observed previously;^35^ both SWNTs and MWNTs can be considered one dimensional in this sense. The flat nucleants, in contrast, can organize a plane of the crystal structure. The ferritin molecules imaged in Fig. 3 appear to be plausible candidates for potential nuclei. The TEM study supports the hypothesis that a low grafting ratio and heterogeneous surface characteristics promotes greater stabilization of protein nuclei for crystallization.

## Conclusion

Here, reduction chemistry was successfully utilised to functionalize an array of carbon nanomaterials of varying geometry with mPEG to produce flat (FLG, GNP), cylindrical (NTs), and spherical (CB) materials. Successful grafting was established by TGA-MS, supported by histograms of the Raman D/G ratio, as well as the observation of increased dispersibility and dispersion stability. The extension of reduction chemistry to the grafting of PEG provides a range of water compatible materials which have potential for many useful applications. The preparation of comparable water compatible CNMs with different geometries is useful for further systematic studies in many contexts from fundamental investigations to applications such as conductive inks, filters and electrodes. Whilst, the chemistry appears broadly similar in all cases, with a degree of grafting related to the packing of the PEG coils on the exposed surface area, the utility of the products varies. The concept of a comparative panel of geometries should inspire a range of fundamental studies to better understand carbon nanomaterials chemistry and to select the best type for specific applications, both important open questions in the field.

The study has specifically shown that PEGylated CNMs can act as effective nucleants for three dimensional protein crystals, when compared to both as-received CNMs and mPEG homopolymer, as well as other commercial nucleants; however the 2D-platelet systems, FLG and GNP functionalized with mPEG, were most successful, consistently producing single crystals for a range of proteins (both models and targets). These types of crystals are suitable for structural determination by X-ray diffraction. TEM investigations of ferritin on FLG-mPEG strongly indicate that the proteins nucleate from both stacked/folded edges and in domains on the basal plane surface; the combination of a large flat surface area, with heterogeneous surface chemistry and topography seems to be especially effective. Direct visualization of the early stages of protein nucleation on these electron transparent nucleants offers exciting opportunities for further fundamental studies. Surface functionalization not only enables the nucleation but stabilises CNM dispersions in water. Compared with existing protein nucleation methods such as seeding, the ability to readily disperse and dispense the nucleants in liquid phase, in order to reproducibly crystallize proteins at low concentrations, is very attractive. It is particularly useful for target protein trials as usually only a very small amount of purified protein is available. In principle, the density and size of the grafted domains may be used to adjust nucleant behaviour, especially as polymer radius of gyration appears to be a key factor in controlling grafting density. The CNM chemistry developed over the past decade, now offers many opportunities for the development of new materials for use in future protein crystallization studies, where more hydrophobic or specific interactions are critical, for example, with membrane proteins, which are notoriously difficult to crystallize.

## Supplementary Material

Supplementary information
