# Supplementary material for: Reductively PEGylated carbon nanomaterials and their use to nucleate 3D protein crystals: a comparison of dimensionality
Source: Chem Sci. 2016 Jan 29;7(4):2916–23. doi: 10.1039/c5sc03595c (PMC6054039; doi:10.1039/c5sc03595c)
Supplement: Supplementary file 1 [file SC-007-C5SC03595C-s001.pdf]

Electronic Supplementary Material (ESI) for Chemical Science.
This journal is © The Royal Society of Chemistry 2016

Supporting Information for

Reductively PEGylated carbon nanomaterials and their use to nucleate 3D protein crystals: a comparison of dimensionality

Hannah S. Leese[‡a], *Lata Govada*[‡b], *Emmanuel Saridakis*[c], *Sahir Khurshid*[b], *Robert Menzel*[a§], *Takuya Morishita*[ad], *Adam J. Clancy*[a], *Edward. R. White*[a], *Naomi E. Chayen*[b]* *and Milo S. P. Shaffer*[a]*

[a] *Department of Chemistry, Imperial College London, London SW7 2AZ, UK.*

[b] *Computational and Systems Medicine, Department of Surgery and Cancer, Imperial College London, London SW7 2AZ, UK.*

[c]*Laboratory of Structural and Supramolecular Chemistry, Institute of Nanoscience and Nanotechnology, National Centre for Scientific Research 'Demokritos' Athens, Greece*

[d] *Toyota Central R&D Labs., Inc., Nagakute, Aichi 480-1192, Japan.*

[§]*School of Chemistry, University of Leeds,*

[*]*email* m.shaffer@imperial.ac.uk, n.chayen@imperial.ac.uk

[‡]contributed equally

Additional Figures and Discussions

Table of Contents

Experimental details……………………………………………………………………………S1

Figure S1 a) TGA-MS weight loss profiles of MWNT-mPEG and MWNT control (polyethylene glycol dimethyl ether), illustrating the determination of the grafting ratio by the difference in weight loss differences and b) TGA-MS of mPEG-Br control……………………………………………… S5

Figure S2 TGA-MS of FLG-mPEG which shows PEG and solvent peaks at the initial wt. % drop at 220°C and PEG fragments at the higher temperature second wt. % loss…………………………… S7

Figure S3 a) Averaged Raman spectra of as received and functionalized CNMs. Note the defective nature of the CB spectra, which lack a 2D peak, have very broad D & G peaks, and high noise b) Single point Raman spectrum of FLG-mPEG, highlighting the strong 2D peak…………………… S8

Figure S4 AFM of few layer graphite particles with height trace…………………………………… S8

Figure S5 UV-Vis plot of FLG-mPEG in water, digital images of FLG-mPEG solutions in water at 1 day and a UV-Vis time plot of FLG-mPEG in water (data recorded in sections due to equipment limitation). Concentration calculated using extinction coefficient 2460 $L\ g^{-1}\ m^{-1}$ from literature.[4] … S9

Figure S6 TGA plots of five newly synthesised samples of FLG-mPEG (newly synthesised mPEG-Br grafted onto freshly prepared reduced FLG). First solvent step removed…………………………… S9

Figure S7 Optical and polarised light microscopy of protein crystals obtained using FLG-mPEG...S10

Figure S8 TEM images of FLG (not PEGylated) and ferritin control. Clusters of ferritin are observed at edges (circled) but not on the surface……………………………………………………………S11

Materials

Poly(ethylene glycol) methyl ether (mPEG), M_W 5000 and poly(ethylene glycol) dimethyl ether, anhydrous THF were all obtained from Sigma-Aldrich. THF was degassed via a freeze-pump-thaw method and dried over 20 wt%, molecular sieves 4Å. Sodium (99.95%, ingot, No. 262714) and naphthalene (99%) were purchased from Sigma-Aldrich and dried under vacuum with phosphorous pentoxide.

Preparation of brominated mPEG. The preparation of mPEG-Br was adapted from previous methods.[1] Poly(ethylene glycol) methyl ether (mPEG) M_W 5000 and 1 eq. of triphenylphosphine (PPh_3) and 4 eq. of tetrabromomethane (CBr_4) were refluxed in dry dichloromethane (DCM) for 24 hr under N_2. The solution was then dried over vacuum. The aqueous phase was then extracted with three portions of CH_2Cl_2 of 45 mL, and the combined extracts were dried over Na_2SO_4. 2g silica was added to the dichloromethane solution and stirred for 2 h in order to eliminate traces of triphenylphosphine oxide remaining after the extraction step. After removal brominated mPEG was precipitated into cold ether (– 78°C), the mPEG-Br was then filtered using a Buchner funnel and washed with cold ethanol. ^1H NMR (DMSO-d_6), δ (ppm): 3.38 (CH_3O–, 3H), 3.68 (PEG main chain): the disappearance of triplet at 4.56 ppm (proton of the hydroxy group of mPEG) and reported values are consistent with previous reports[1], and indicates quantitative conversion of mPEG-Br.

Reduction and Functionalization of Carbon Nanomaterials. Reduction chemistry was utilized to produce nanotubides, graphenides and reduced carbon black solutions using Na/naphthalene with either DMAc or THF. A typical experiment for the preparation of graphenides/nanotubides/and reduced carbon black involved heating the carbon nanomaterial to 400°C under vacuum ($<10^{-1}$ mbar) for 1 hr. The Na was used as received from Sigma Aldrich. The naphthalene, purchased from Sigma Aldrich, was dried overnight in a vacuum oven with phosphorous pentoxide before transferring to a N_2 filled glove box. All samples were prepared in a N_2 filled glove box. 1 mmol of sodium and 1 mmol of naphthalene were added to 10 ml of degassed THF or DMac and stirred for 1 day. A dark green color was observed. 1 mL of the Na/Naphthalene solution was added to dry nanocarbon and more degassed THF or DMAc was added to adjust concentration. The graphene/sodium naphthalide solution was sonicated for 15 minutes and stirred for 1 day before slowly adding mPEG-Br to the mixture and stirred. The solution was removed from the glove box after 24 h

and quenched with zero grade O_2. After bubbling O_2 (0.05-0.1 cm^3 min^{-1}) into the solution, it was stirred overnight under O_2 for the oxidation of any remaining charges on functionalized nanocarbons. More THF or DMAc was added and then ethanol (10 ml) was added slowly followed by water (20 ml). The mixture was filtered using Millipore Omnipore PTFE 100 nm membrane filters, washing thoroughly with THF, ethanol and water. After washing the sample with THF, ethanol and water again, the product was obtained after drying overnight under vacuum at 80 °C.

Characterization of PEGylated Carbon Nanomaterials. In order to thoroughly characterize the functionalized materials several techniques were utilized. Transmission electron microscopy (TEM) was carried out using a JEOL2010 TEM at 200 kV operating voltage. The TEM of ferritin and FLG-mPEG was conducted on the Titan 80/300 TEM/STEM at 80 kV. Samples were typically prepared on 300 copper mesh holey carbon grids (Agar Scientific) by drop-casting onto the grid, supported by filter paper and left to dry before putting under vacuum. Atomic force microscopy (AFM) was carried out using a Nanoscope IV Digital Instruments AFM (Veeco). Nanosensor tapping mode probes supplied by Windsor Scientific were used. Samples were typically prepared by drop casting a dispersion of the functionalised carbon material on a clean silicon wafer. Thermogravimetric mass spectrometry analysis (TGA-MS) was carried out using a METTLER TGA/DSC1 integrated with a Hiden HPR–20 QIC R&D mass spectrometer. Samples were heated to 100 °C, under a N_2 atmosphere (20 ml min^{-1}) which was held isothermally for 60 minutes to remove residual water and/or solvent; the temperature was increased to 850 °C at a constant ramping rate of 10 °C min^{-1} under flowing N_2 (20 ml min^{-1}). Raman spectra were collected on a Renishaw inVia micro-Raman (1000 – 3000 cm^{-1}), using a 532 nm laser. Point and mapping spectra (between 500 – 1000 spectra over at least 3 different areas) were conducted. Samples were prepared by drop cast dispersions on a glass slide or silicon wafer. The measurements of adsorption and desorption isotherms of nitrogen at 77 K were carried out on 20-50 mg of CNMs using a Micromeritics ASAP 2010 apparatus. Specific surface areas were calculated according to the Brunauer, Emmett and Teller (BET) equation from the adsorption isotherms in the relative pressure range of 0.05–0.20 p/p_0. Prior to analysis, the samples were degassed with continuous N_2 flow at 100 °C for 12 hours.

Crystallization Trials. Thaumatin, lysozyme, trypsin, hemoglobin and catalase were obtained as highly pure lyophilised powders from Sigma-Aldrich, UK. Thaumatin (T-7638)

from *Thaumatococcusdaniellii* was prepared in distilled water at 20 mg ml^{-1}; lysozyme (L-6876) from chicken egg-white was prepared in 50 mM sodium acetate at concentrations of 20 and 40 mg ml^{-1}; trypsin (T9201) from porcine pancreas was prepared in 10 mg ml^{-1} benzamidine hydrochloride, 10 mM calcium chloride and 20 mM HEPES (pH 7.0) at 50 mg ml^{-1}; bovine hemoglobin (H-2500) was prepared in ultrapure water at 45 mg ml^{-1} and catalase (C-9322) from bovine liver was prepared in 10mM HEPES buffer (pH 7.0) at concentration 11 mg ml^{-1}. The anti-CCR5 Fab fragment RoAb13 was given by Professor Benny Chain (University College London) and was prepared in 0.1M sodium chloride and 20 mM HEPES (pH 7.0) at 10 mg ml^{-1}.

All reagents were of analytical grade, obtained commercially. All solutions were freshly prepared, using milli-Q water (Merck Millipore, USA). Salt and buffer stock solutions were kept at room temperature for the duration of the study. Polyethylene glycols (PEG) of various mean molecular weights, namely 2000 monomethyl ether (2K MME - 84797, Sigma-Aldrich, UK); 3350 (88276, FlukaBiochemika); 8000 (P-4463, Sigma-Aldrich, UK) were used to make solutions kept at 4 ºC.

Crystallization experiments were performed by the hanging drop method in EasyXtal Tool plates. 400 µl of the appropriate crystallization condition was dispensed in each reservoir. For each trial, 1 µl of the desired protein solution was mixed with 1 µl of the condition containing the dispersed nucleant (with the exception of the control experiments where 1 µl of the condition was taken from the 400 µl reservoir). To minimise agglomeration, the grafted CNMs were not allowed to dry during work-up, and were used directly as aqueous dispersions. When using the nucleant dispensed in water, 1 µl of the protein solution was mixed with 1 µl of the crystallization condition taken from the 400 µl reservoir and then the nucleant solution was added at a volume which is 10% of the drop volume. These drops were dispensed on a screw cap lid, which was then inverted and sealed over the reservoir. The drops were incubated at 20°C and monitored every 24 h for 4 weeks. The nucleant trials were repeated at least 3 times. The controls were dispensed on the same caps as their respective test drops and sealed over the same well. All crystallization conditions are shown in Table S2.

Determination of PEGylated carbon nanomaterial grafting ratios, using PEGylated MWNT as an example:

a)

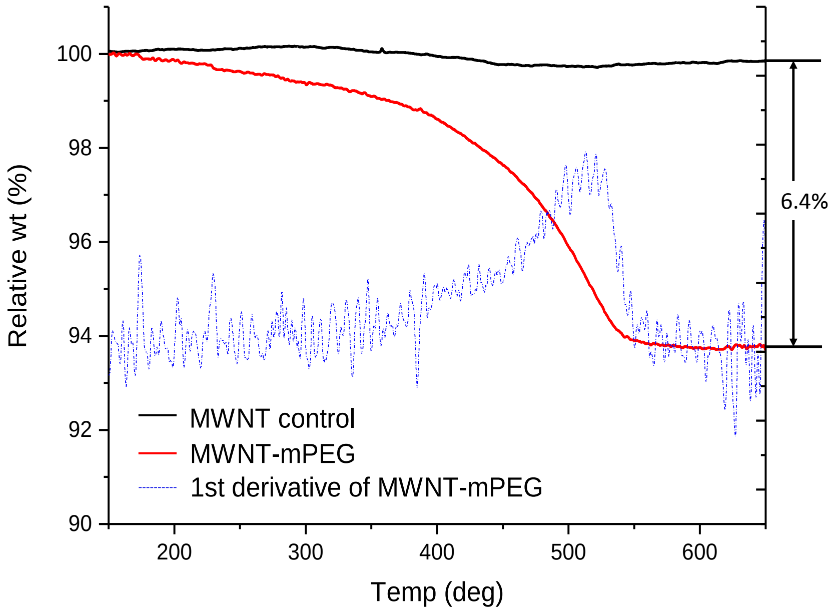

b)

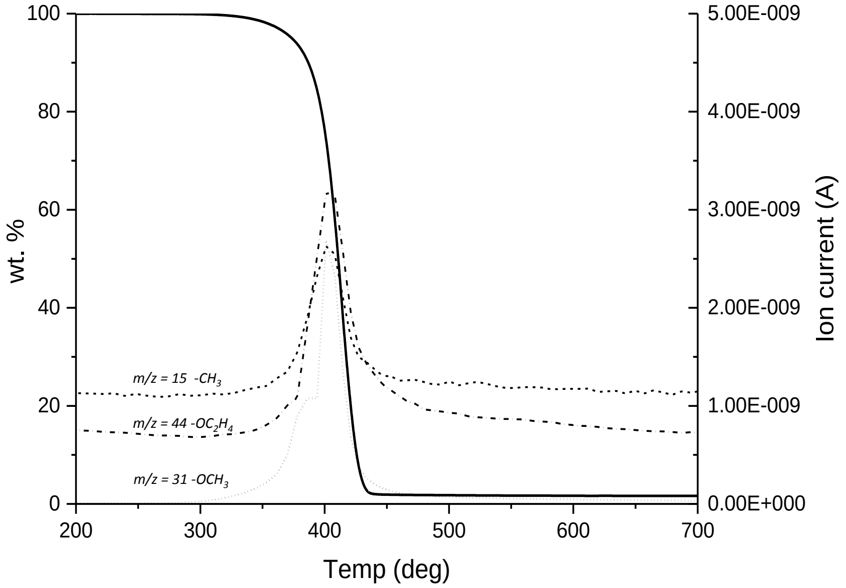

Figure S1 a) TGA-MS weight loss profiles of MWNT-mPEG and MWNT control (polyethylene glycol dimethyl ether), illustrating the determination of the grafting ratio by the difference in weight loss and b) TGA-MS of mPEG-Br control

The grafting ratio (GR = mass fraction of grafted organic relative to the original carbon framework) was deduced, taking the residue determined by TGA to be zero for PEG and the mass fraction remaining of the carbon framework in the grafted samples to be the same as the relevant control. Specifically, the difference between the control residue, R_c, and the sample residue, R_S, gives GR as $(R_C\text{-}R_S)/R_S = R_C/R_S - 1$. In the example in Fig S1a, R_C ~ 100%, R_S = 93.6%, therefore the grafting ratio GR = 6.4/93.6.

The number of structural nanoparticle carbons per mPEG chain (C: PEG ratio, 'grafted stoichiometry') can be estimated from:

$$\frac{1}{\left(\frac{GR_{PEG}}{M_W\text{mPEG}} M_w C\right)} = \text{C: PEG}$$

where GR_{PEG} is the grafting ratio of PEG, and M_WmPEG and $M_w C$ are the molar weights of the mPEG and carbon, respectively.

The 'Surface Concentration of Grafted PEG', the molar concentration of grafted PEG chains per unit surface area of carbon (mol m^{-2}) was determined by first estimating the number of PEG chains per gram of carbon ('#PEGs per g of carbon' in Table S1) from the grafting ratio and the mass of a mPEG chain (= GR_{PEG} / 8.3×10^{-21}), then normalising by the BET surface area (SA; reported in Table 1 and S1):

$$[Grafted\ PEG] = \frac{\left(\#PEGs/_{SA}\right)}{N_A}$$

Calculation of polymer spacing

de Gennes' theory of polymer conformation at a surface compares chain separation and the Flory dimension R_F,[2,3] in order to assess the likely polymer conformation. PEG separation D shown in Table 1 and S1 were estimated from the square root of the average area occupied by a PEG chain on the carbon surface ($A = 1 / ([Grafted\ PEG]*N_A)$).

Table S1 Summary of calculated values for chain separation

Nucleant Material	Surface area (m^2 g^{-1})	Average Pore Size (nm)	GR (wt% of PEG)	#PEGs per g of carbon	*A* occupied by 1 PEG chain (m^2)	PEG separation, *D* (nm)	Surface conc. of Grafted PEG (μmolm^{-2})
MWNT	180	12.6	6.8±1.5	8.23×10^{18}	2.18×10^{-17}	5.3	0.076
SWNT	670	6.4	12.6±3.2	1.52×10^{19}	4.41×10^{-17}	7.5	0.038
FLG	680	-	10.6±1.1	1.28×10^{19}	5.32×10^{-17}	8.2	0.031
GNP	680	-	11.0±2.2	1.32×10^{19}	5.14×10^{-17}	8.1	0.032
CB	220	10.3	135.3±6.1	1.63×10^{20}	1.35×10^{-18}	1.3	1.230

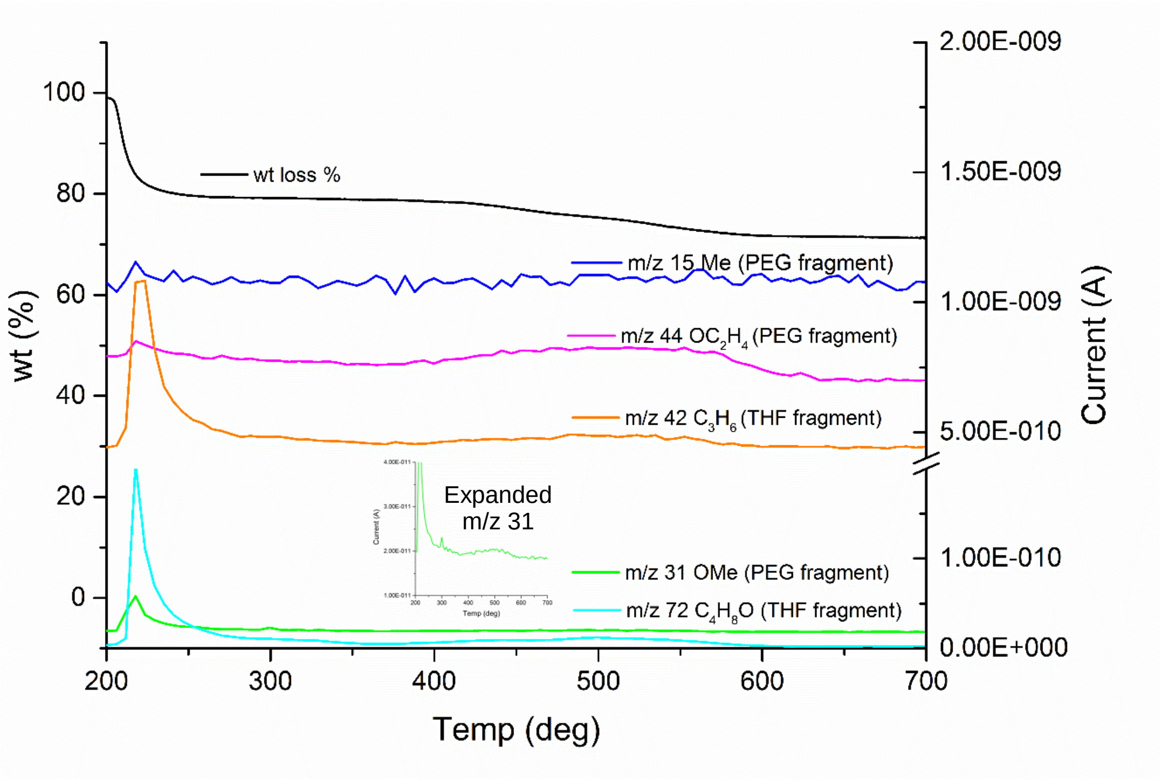

Figure S2 TGA-MS of FLG-mPEG which shows PEG and solvent peaks at the initial wt. % drop at 220°C and PEG fragments at the higher temperature second wt. % loss.

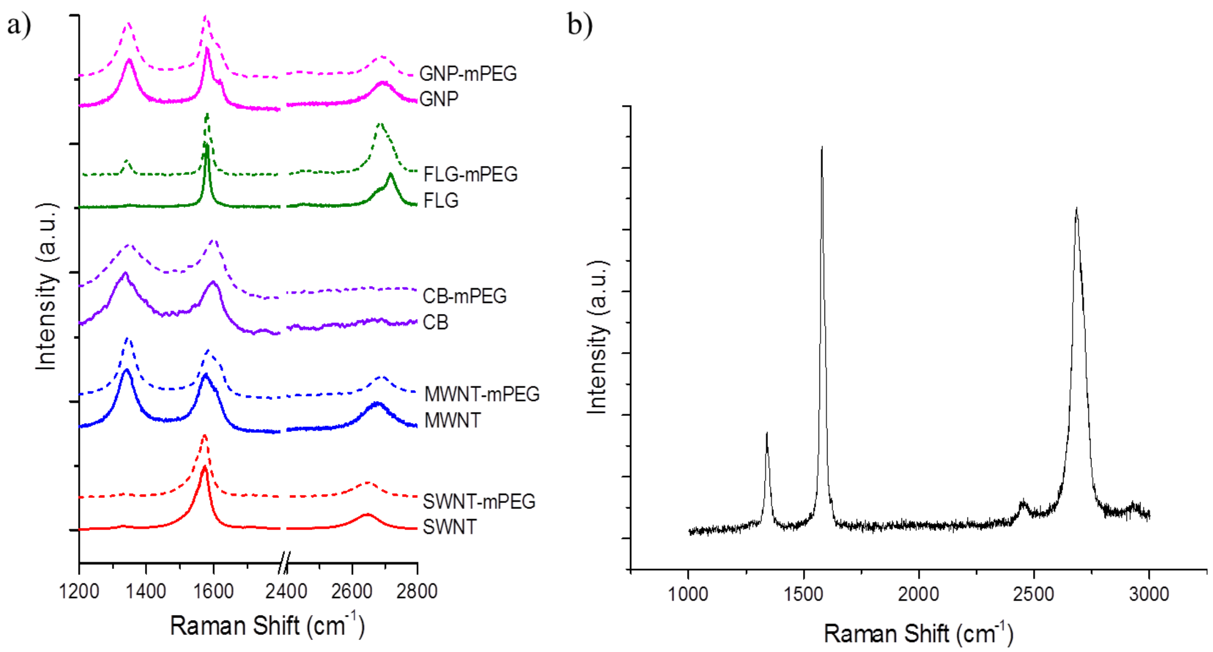

Figure S3 a) Averaged Raman spectra of as received and functionalized CNMs. Note the defective nature of the CB spectra, which lack a 2D peak, have very broad D & G peaks, and high noise b) Single point Raman spectrum of FLG-mPEG, highlighting the strong 2D peak.

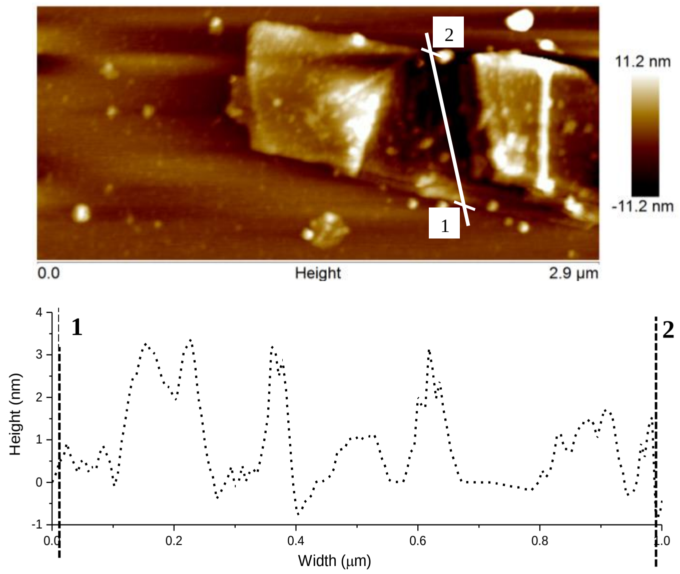

Figure S4 AFM of FLG-mPEG particles with height trace.

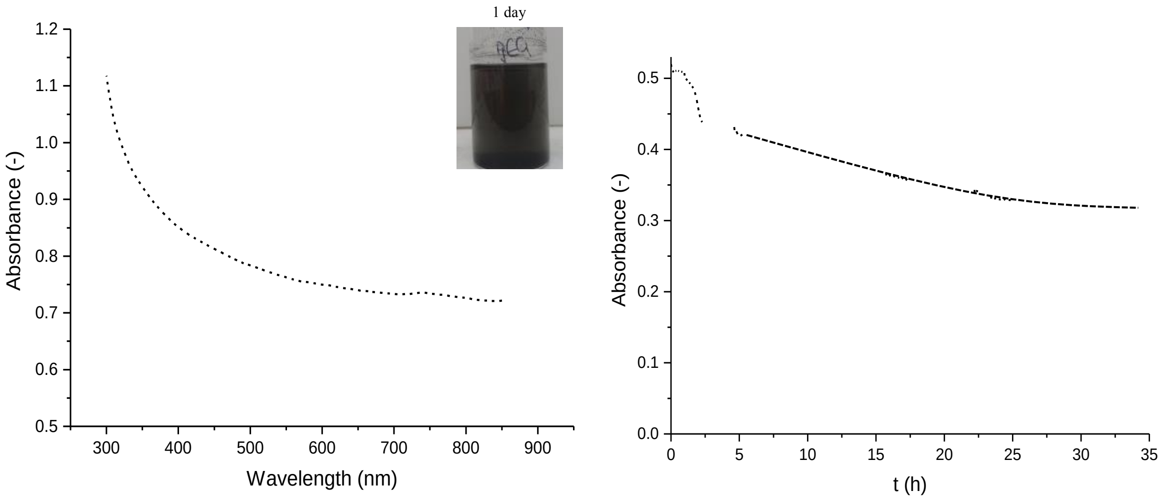

Figure S5 UV-Vis plot of FLG-mPEG in water, digital images of FLG-mPEG solutions in water at 1 day and a UV-Vis time plot of FLG-mPEG in water (data recorded in sections due to equipment limitation). Concentration calculated using extinction coefficient 2460 *L* g^{-1} m^{-1} from literature.[4]

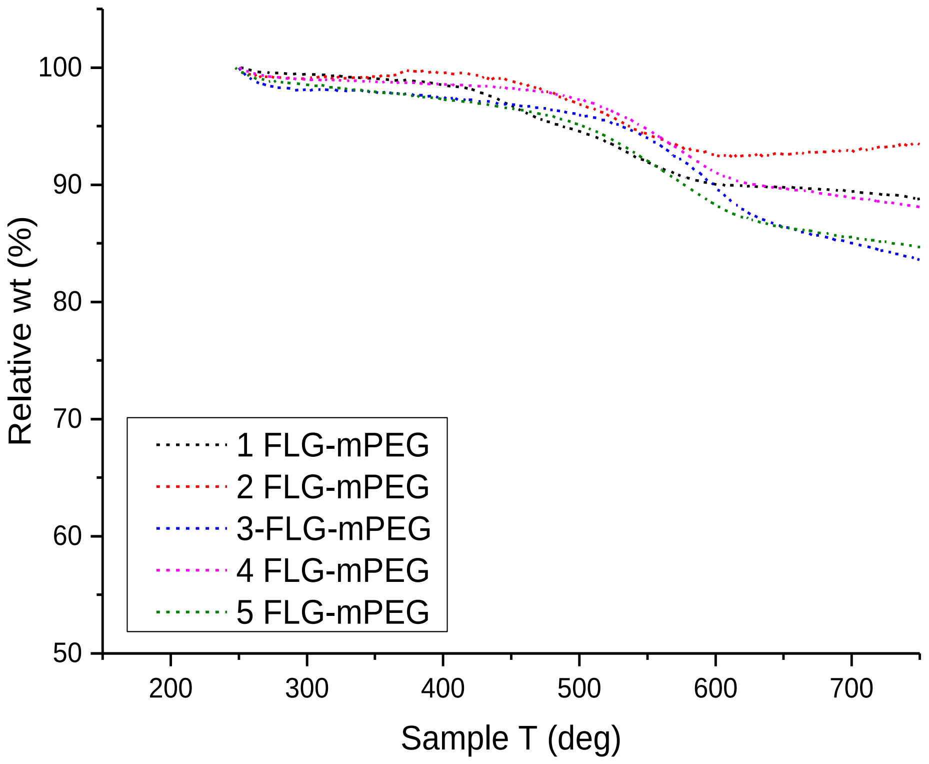

Figure S6 TGA plots of five newly synthesised samples of FLG-mPEG (newly synthesised mPEG-Br grafted onto freshly prepared reduced FLG). First solvent step removed.

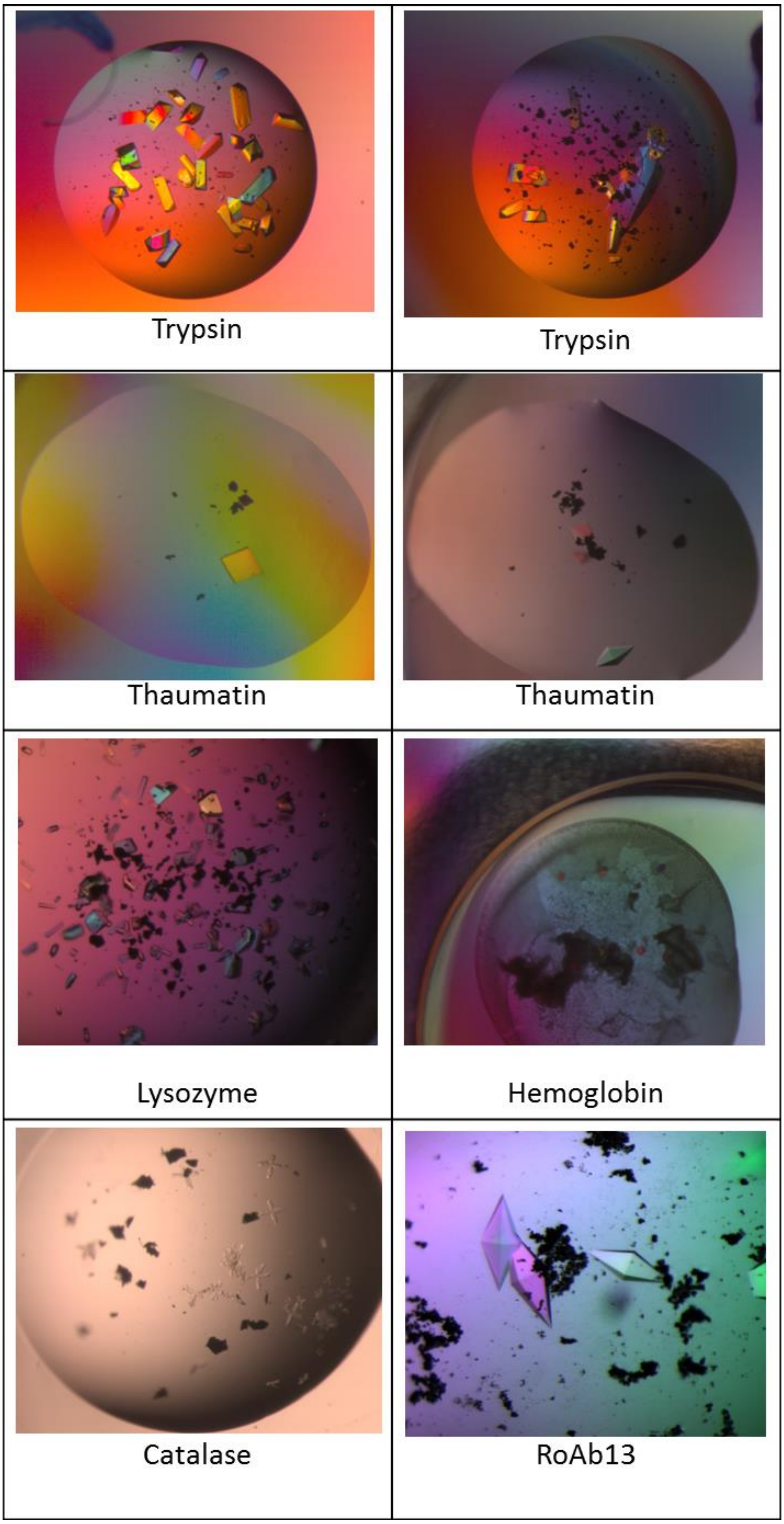

Figure S7 Optical and polarised light microscopy of protein crystals obtained using FLG-mPEG.

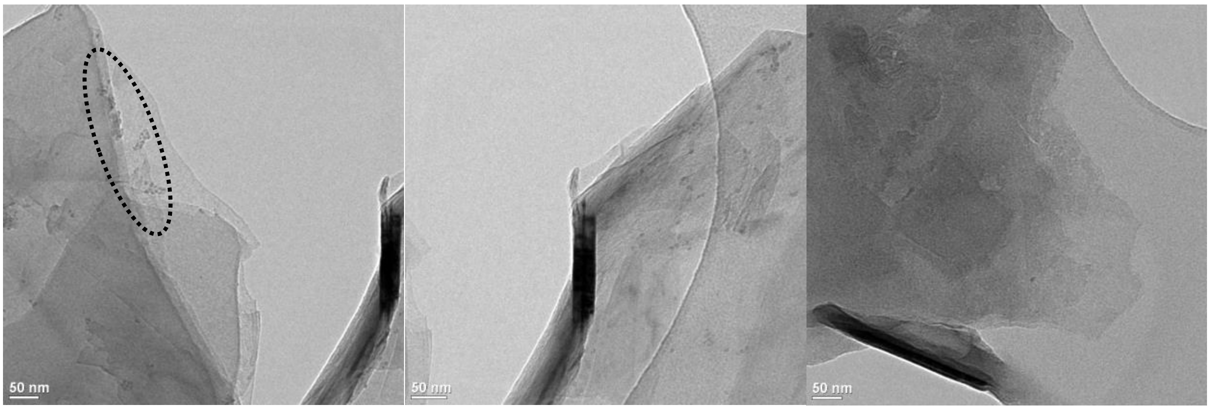

Figure S8 TEM images of FLG (not PEGylated) and ferritin control. Clusters of ferritin are observed at edges (circled) but not on the surface.

Developing the Liquid Nucleant

In order to improve accuracy when dispensing nucleants into protein drops, rather than introducing the nucleants as solid materials they were dispersed into liquids at 50 – 100 μg ml^{-1}. Initially all the nucleants were dispersed by brief (< 1 minute) bath sonication directly into the respective crystallizing agents (Table S2). These liquid nucleants were effective, but to simplify this methodology, a stock solution of the nucleant in water was added to the protein and crystallizing agents; this approach was found to be equally effective.

Table S2 List of protein concentrations and crystallisation conditions

Protein	Conc. mg ml^{-1}	Crystallization Conditions
Lysozyme	20	0.1 M sodium acetate at pH 4.5 and 0.5-1.5M NaCI
Thaumatin	20	0.1 M Bis-tris propane at pH 6.8and 0.1-1M Na/K tartrate (NaKT)
Trypsin	50	0.2 M ammonium sulphate, 0.1M cacodylate at pH 6.5 and 5-30%(w/v) PEG 8000
Catalase	11	0.1 M tri-ammonium citrate at pH 6.8 and 10-20%(w/v) PEG 3350
Hemoglobin	45	20%–25% (w⁄v) PEG 3350, 0.2 M $MgCl_2$ and 0.1 M Bis-Tris buffer, pH 5.5.
RoAb13	10	10 mM nickel chloride, 0.1 M TRIS at pH 8.5 and16%-20 %(w/v) PEG2K MME

References

1. Y.-J. Zhao, Y.-Q. Zhai, Z.-G. Su, G.-H. Ma, *Polymers for Advanced Technologies*, 2010, **21**, 867–873

2. P. G. de Gennes, *Macromolecules*, 1980, **13**, 1069–1075.

3. C. H. J. Choi, J. E. Zuckerman, P. Webster and M. E. Davis, *Proceedings of the National Academy of Sciences*, 2011, **108**, 6656–6661.

4. Y. Hernandez, V. Nicolosi, M. Lotya, F. M. Blighe, Z. Sun, S. De, I. T. McGovern, B. Holland, M. Byrne, Y. K. Gun'Ko, J. J. Boland, P. Niraj, G. Duesberg, S. Krishnamurthy, R. Goodhue, J. Hutchison, V. Scardaci, A. C. Ferrari and J. N. Coleman, *Nat Nano*, 2008, **3**, 563–568.
